# A Cell Permeable NPE Caged ADP-Ribose for Studying TRPM2

**DOI:** 10.1371/journal.pone.0051028

**Published:** 2012-12-07

**Authors:** Peilin Yu, Qian Wang, Li-He Zhang, Hon-Cheung Lee, Liangren Zhang, Jianbo Yue

**Affiliations:** 1 Department of Physiology, University of Hong Kong, Hong Kong, China; 2 State Key Laboratory of Natural and Biomimetic Drugs, School of Pharmaceutical Sciences, Peking University, Beijing, China; 3 Department of Toxicology, School of Public Health, Zhejiang University, Hangzhou, China; University of Oldenburg, Germany

## Abstract

Transient potential receptor melastatin-2 (TRPM2) is a non-selective Ca^2+^-permeable cation channel of the TRPM channel subfamily and is mainly activated by intracellular adenosine diphosphate ribose (ADPR). Here we synthesized a 1-(2-nitrophenyl)ethyl caged ADPR (NPE-ADPR) and found that uncaging of NPE-ADPR efficiently stimulated Ca^2+^, Mg^2+^, and Zn^2+^ influx in a concentration-dependent manner in intact human Jurkat T-lymphocytes. The cation influx was inhibited by inhibitors or knockdown of TRPM2. Likewise, uncaging of NPE-ADPR markedly induced cation entry in HEK 293 cells that overexpress TRPM2. As expected, high temperature increased the ability of the photolyzed NPE-ADPR to induce cation entry, whereas acidic pH inhibited. Moreover, the absence of extracellular Ca^2+^ significantly inhibited Mg^2+^ and Zn^2+^ influx after uncaging NPE-ADPR. On the other hand, the absence of extracellular Na^+^ or Mg^2+^ had no effect on photolyzed NPE-ADPR induced Ca^2+^ entry. Taken together, our results indicated that NPE-ADPR is a cell permeable ADPR analogue that is useful for studying TRPM2-mediated cation entry in intact cells.

## Introduction

TRPM2 is a non-selective cation channel that is Ca^2+^ permeable. It has six transmembrane domains, and is best known as a ‘chanzyme’ due to its function as both ion channel and pyrophosphatase. The pyrophosphatase (Nudix-like) domain of TRPM2 is located at its C-terminus, and a calmodulin binding IQ-like motif is located at its N-terminus. The pore forming region of TRPM2 sits between the 5th and 6th transmembrane domains with the N- and C-termini lying in the cytoplasm. TRPM2 in some tissues is expressed in multiple isoforms, yet the significance of these isoforms remains to be determined [1]–[3]. Although TRPM2 is primarily located at the plasma membrane, it has also been detected on lysosomes, possibly acting as a Ca^2+^ releasing channel in the acid Ca^2+^ store [4].

TRPM2 mediated Ca^2+^ influx has been indicated in several physiological and pathophysiological processes, including insulin secretion, pro-inflammatory cytokine production, permeability of endothelium, and dendritic cell maturation and chemotaxis [4]–[11]. Since TRPM2 can be activated by oxidative stress, it has recently emerged as a potential therapeutic target in fighting oxidative stress-related diseases, including diabetes, inflammation, myocardial infarction, and neurodegenerative diseases [12]–[16]. In addition, genetic variants of the TRPM2 gene have been associated with Western Pacific amyotrophic lateral sclerosis, parkinsonism-dementia, and bipolar disorders [17]–[20].

The most potent and primary intracellular activator for TRPM2 is adenosine diphosphate ribose (ADPR) via its Nudix-like domain [21]. Intracellular ADPR can be generated from the hydrolysis of NAD^+^ by glycohydrolases, e.g., the mitochondrial NADase and CD38, in response to a wide variety of physiological stimuli, including oxidative and nitrosative stress, beta amyloid, and tumor necrosis factor [11],[22]–[24]. ADPR can also be generated in the nucleus by the sequential action of poly-ADPR polymerases and poly-ADPR glycohydrolases that are triggered by DNA damage [25],[26]. On the other hand, adenosine monophosphate (AMP), generated from hydrolysis of ADPR by TRPM2's pyrophosphatase activities, is a potent inhibitor of TRPM2, constituting a negative feedback loop to shut down the activation of TRPM2 by ADPR [27]. In addition, cell stress can activate Sir2 deacetylases to produce 2′-*O*-acetyl-ADPR, which can directly gate TRPM2 for Ca^2+^ influx [28],[29]. NAADP or cADPR, the other two products of CD38, can also either directly or in synergy with ADPR activate TRPM2 [30]. The gating of TRPM2 is influenced by [Ca^2+^]_i_, temperature, and pH as well [8],[31]–[35].

Given the importance of TRPM2 in diverse cellular processes and ADPR as its main activator, it is surprising that few ADPR analogues have been synthesized. Thus far, the studies on TRPM2 were mainly done by patch clamp recording with dialyzed ADPR. Here we synthesized a 1-(2-nitrophenyl)ethyl caged ADPR (NPE-ADPR) and found that uncaging of NPE-ADPR induced multiple cation entry in intact human Jurkat and HEK293 cells via TRPM2.

## Results

### Synthesis And Purification Of Npe-Adpr

**Figure 1:**
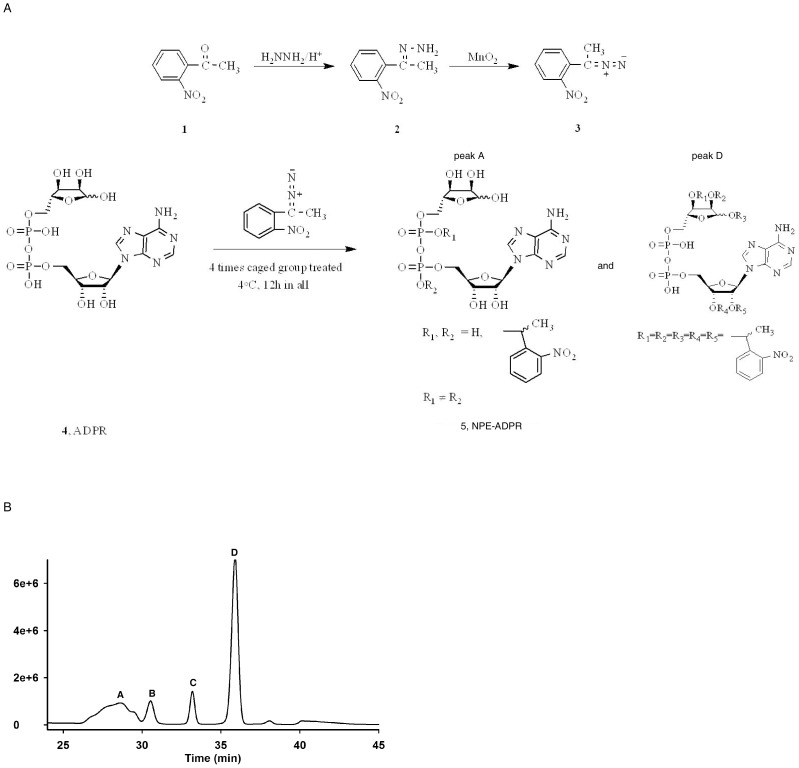
Synthesis and purification of 1-(2-nitrophenyl)ethyl (NPE)-ADPR. (**A**) Synthesis route of NPE-ADPR. (**B**) HPLC purification of NPE-ADPR.

As the main intracellular activator of TRPM2, ADPR is hydrophilic and cannot cross the plasma membrane. Thus far, the studies on TRPM2 were mainly performed by patch clamp recording in single cells. Therefore, cell-permeant ADPR analogues should be valuable research tools in dissecting the mechanism of TRPM2-induced cation entry. We reasoned that adding a caged group to one of the phosphates on ADPR could increase its membrane permeability and enable it to accumulate inside cells. Photolysis by UV can then release the bioactive ADPR to activate TRPM2 for cation entry, thereby providing more precise control in studying the TRPM2/ADPR signaling in multiple cells simultaneously. We screened a series of caged groups, and found that only the 1-(2-nitrophenyl)ethyl (NPE) caged group can be successfully linked to one of the phosphates of ADPR (Figure 1A). Analyses by HPLC showed that the reaction mixture contained four peaks (Figure 1B). By mass spectrometry and NMR analyses, we found that peak A contained the predicated NPE caged ADPR isomers with one NPE coupled to one of the phosphates, whereas peak D contained an unexpected NPE-ADPR product with five NPE groups reacted with all the hydroxyl groups in the riboses. Peaks B and C did not contain any ADPR related products.

**Figure 2:**
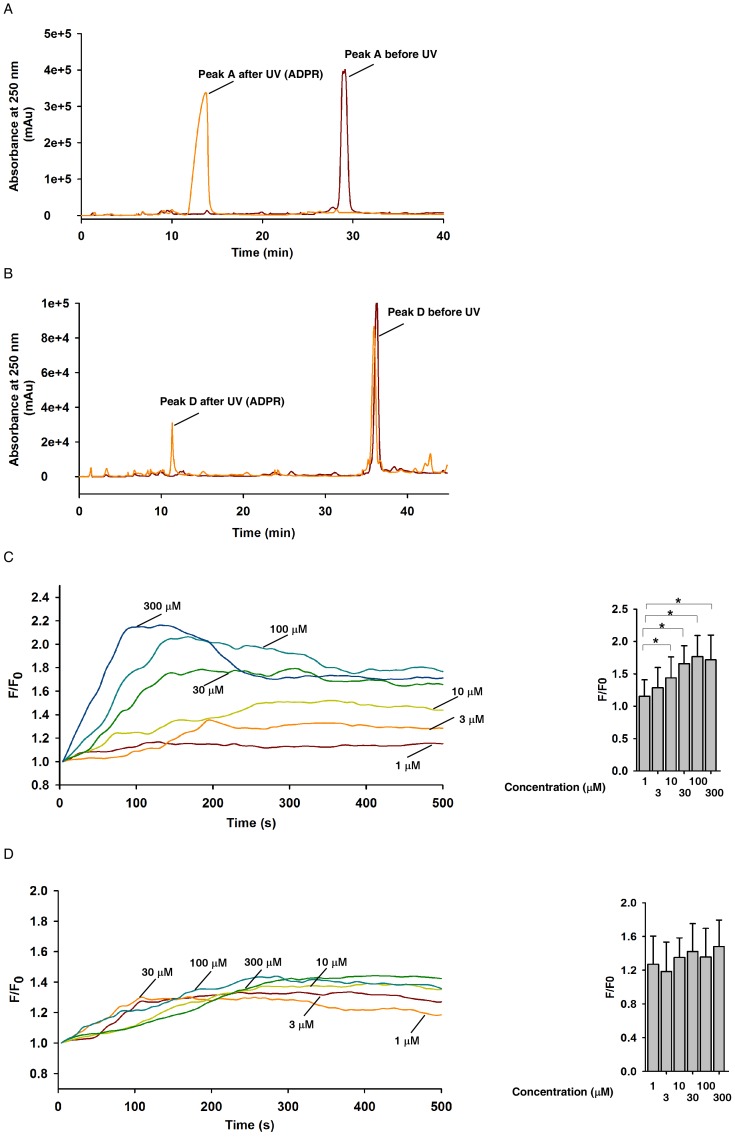
Characterization of NPE-ADPR. (**A**) and (**B**) HPLC analysis of Peak A (A) and peak D (B) before and after UV photolysis. (**C**) and (**D**) The concentration response curve of Ca^2+^ increase in human Jurkat cells induced by peak A (C) and peak D (D) after UV photolysis. Fluo-4 loaded cells were incubated in regular HBSS containing extracellular Ca^2+^ during the experiment. Data quantifications of [Ca^2+^]_i_ peak induced by drug treatment in (C) and (D) were expressed as mean ± S.D., n = 30–40 cells. The * symbols indicate the results of *t* Test analysis, *p*<0.05, compared with cells treated with 1 µM NPR-ADPR. In (C) and (D), cells were all continuously incubated with NPE-ADPR, and UV photolysis started at the beginning of the measurement and was repeated every 7 second throughout the experiments.

Purified peak A and peak D were then subjected to photolysis by UV illumination, and subsequently analyzed by HPLC. After UV activation, only peak A was efficiently uncaged to generate free ADPR (Figure 2A), whereas very little of peak D was photolyzed to free ADPR (Figure 2B), indicating that the bond between NPE and the hydroxyl groups of the riboses is stable.

### Uncaged Npe-Adpr Induction Of Ca^2+^ Influx

Next, we examined whether peak A or peak D could induce intracellular Ca^2+^ increases in human Jurkat T cells after UV illumination. Fluo-4 loaded cells were incubated with NPE-ADPR for 5 min and then subjected to Ca^2+^ measurement without removing the compound in the medium. As shown in Figure 2C, peak A induced cytosolic Ca^2+^ ([Ca^2+^]_i_)increases in Jurkat cells in a concentration dependent manner after UV photolysis. On the other hand, peak D only marginally induced [Ca^2+^]_i_ changes independent of the concentration after UV activation (Figure 2D), again confirming that the bond between NPE and the hydroxyl groups of the riboses is resistant to UV photolysis. In addition, peak A did not evoke any Ca^2+^ changes in Jurkat cells without UV uncaging, and UV illumination in the absence of NPE-ADPR also failed to induce Ca^2+^ (Figure S1). Therefore, we only characterized the properties of peak A, named as NPE-ADPR, in the later experiments.

**Figure 3:**
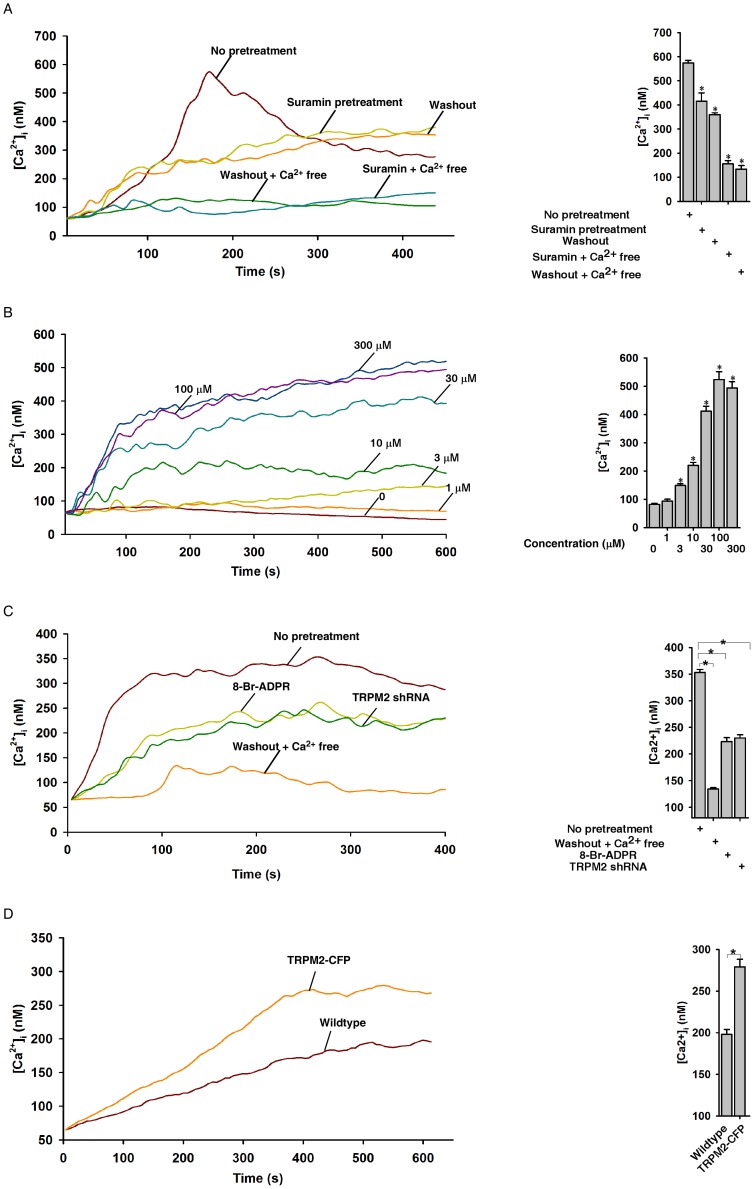
Characterization of the NPE-ADPR-induced Ca^2+^ increase. (**A**) The photolyzed NPE-ADPR (30 µM)-induced Ca^2+^ increases in Fluo-4 loaded human Jurkat cells were inhibited by suramin or removal of extracellular Ca^2+^ (washout), and was completely abolished by combination of removal of extracellular Ca^2+^ and extracellular NPE-ADPR or by combination of removal of extracellular Ca^2+^ and suramin pretreatment. The * symbols indicate the results of *t* Test analysis, *p*<0.05, compared with cells without pretreatment. (**B**) Uncaging of intracellular NPE-ADPR triggered Ca^2+^ increases in a concentration dependent manner in Fluo-4 loaded human Jurkat cells in regular HBSS containing extracellular Ca^2+^. The * symbols indicate the results of *t* Test analysis, *p*<0.05, compared with cells loaded with buffer only. (**C**) Pretreatment of Jurkat cells with 8-Br-ADPR (100 µM) or knockdown of TRPM2 significantly inhibited the photolyzed NPE-ADPR (30 µM) induced Ca^2+^ influx in Fluo-4 loaded Jurkat cells. The * symbols indicate the results of *t* Test analysis, *p*<0.05, compared with cells without pretreatment. (**D**) Uncaging of NPE-ADPR (30 µM) induced Ca^2+^ influx in Fluo-4 loaded HEK293 cells that transiently express TRPM2-CFP. The * symbols indicate the results of *t* Test analysis, *p*<0.05. Data quantifications of [Ca^2+^]_i_ peak induced by drug treatment in (A), (B), (C), and (D) were expressed as mean ± S.E., n = 30–40 cells. In (B), (C), and (D), after Fluo-4 loaded cells were incubated with NPE-ADPR, extracellular NPE-ADPR was then removed before UV photolysis to start Ca^2+^ measurement.

We traced the sources of the [Ca^2+^]_i_ increases induced by uncaging NPE-ADPR. Since external ADPR induced [Ca^2+^]_i_ increases in Jurkat cells (Figure S2) and other cell types [36], we pretreated Jurkat cells with suramin [337], a potent P2Y receptor blocker, to eliminate the effects of extracellular ADPR on [Ca^2+^]_i_. Indeed, suramin pretreatment markedly inhibited the photolyzed NPE-ADPR from inducing [Ca^2+^]_i_ increases, in which the pattern of Ca^2+^ changes resembled that observed in the absence of extracellular NPE-ADPR (Figure 3A). Furthermore, removal of both extracellular Ca^2+^ and NPE-ADPR abolished the photolyzed NPE-ADPR from inducing [Ca^2+^]_i_ increases. Similarly, the combination of suramin pretreatment and removal of extracellular Ca^2+^ prevented the photolyzed NPE-ADPR from inducing [Ca^2+^]_i_ increases as well (Figure 3A). These data indicated that NPE-ADPR can enter intact Jurkat cells and trigger Ca^2+^ influx after UV photolysis. In the later experiments, we mainly studied the effects of intracellular NPE-ADPR by removing the extracellular compound through cell washing after loading.

After removal of extracellular NPE-ADPR, uncaged NPE-ADPR still induced [Ca^2+^]_i_ increases in a concentration dependent manner in Jurkat cells, albeit at lesser extent than without washing (Figure 3B). Moreover, pretreatment of cells with the TRPM2 antagonist, 8-Br-ADPR [38], or knockdown of TRPM2 [39] in Jurkat cells significantly inhibited the photolyzed NPE-ADPR-induced [Ca^2+^]_i_ increases (Figure 3C). Similarly, in the presence of suramin, pretreatment with 8-Br-ADPR or after TRPM2 knockdown, the photolyzed NPE-ADPR induced [Ca^2+^]_i_ increases in Jurkat cells was abolished (Figure S3). Likewise, uncaging of NPE-ADPR markedly induced Ca^2+^ entry in HEK 293 cells that overexpress TRPM2 (Figure S4) as compared to that in wildtype cells (Figure 3D). Taken together, these data indicate that uncaging of intracellular NPE-ADPR induces Ca^2+^ influx via TRPM2.

### Uncaged Npe-Adpr Induction Of Mg^2+^ Influx

**Figure 4:**
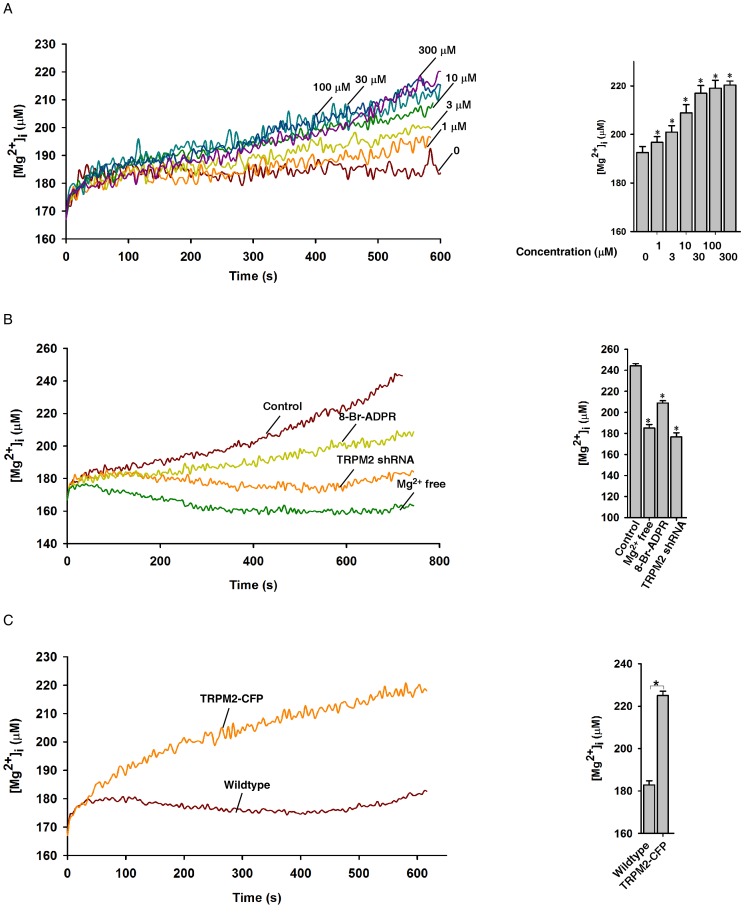
Characterization of the NPE-ADPR induced Mg^2+^ influx. (**A**) Uncaging of intracellular NPE-ADPR (30 µM) induced intracellular Mg^2+^ increases in a dose dependent manner in Mag-Fura-2 loaded human Jurkat cells in regular HBSS. The * symbols indicate the results of *t* Test analysis, *p*<0.05, compared with cells loaded with buffer only. (**B**) Pretreatment of Jurkat cells with 8-Br-ADPR (100 µM), or knockdown of TRPM2, or removal of extracellular Mg^2+^ markedly inhibited uncaged NPE-ADPR (300 µM)-induced Mg^2+^ influx in Mag-Fura-2 loaded human Jurkat cells. The * symbols indicate the results of *t* Test analysis, *p*<0.05, compared with cells without pretreatment. (**C**) Uncaging of NPE-ADPR (300 µM) induced Mg^2+^ influx in Mag-Fura-2 loaded HEK293 cells that transiently express TRPM2-CFP. Data quantifications of [Mg^2+^]_i_ peak induced by drug treatment in (A), (B), and (C) were expressed as mean ± S.E., n = 30–40 cells. In (A), (B), and (C), after Mag-Fura-2 loaded cells were incubated with NPE-ADPR, extracellular NPE-ADPR was then removed before UV photolysis to start the Mg^2+^ measurement.

TRPM2 is a non-selective cation channel, and it has been shown previously that ADPR can stimulate Mg^2+^ influx via TRPM2 [40]. We therefore examined the ability of photolyzed NPE-ADPR to induce Mg^2+^ entry in Jurkat cells. Mag-Fura-2 AM was used to measure intracellular Mg^2+^ concentrations. As shown in Figure 4A, uncaging of NPE-ADPR induced intracellular Mg^2+^ increases in a concentration dependent manner in Jurkat cells. In addition, NPE-ADPR did not evoke any Mg^2+^ changes in Jurkat cells without UV uncaging, and UV illumination in the absence of NPE-ADPR also failed to induce Mg^2+^ (Figure S5). Moreover, removal of extracellular Mg^2+^, or treatment with 8-Br-ADPR, or knockdown of TRPM2 abolished the photolyzed NPE-ADPR-induced Mg^2+^ increases (Figure 4B). Likewise, uncaging of NPE-ADPR induced Mg^2+^ entry only in HEK 293 cells that overexpress TRPM2 but not in wildtype cells (Figure 4C). Notably, Mag-Fura-2 is insensitive to Ca^2+^ change, evidenced by the fact that anti-CD3 antibody, OKT3, markedly induced Ca^2+^ increases in Fura-2 loaded Jurkat cells, whereas it failed to induce any fluorescence changes on Maga-Fura-2 loaded cells (Figure S6). Thus, these data demonstrated that uncaging of NPE-ADPR induces Mg^2+^ entry via TRPM2.

### Uncaged Npe-Adpr Induction Of Zn^2+^ Influx

**Figure 5:**
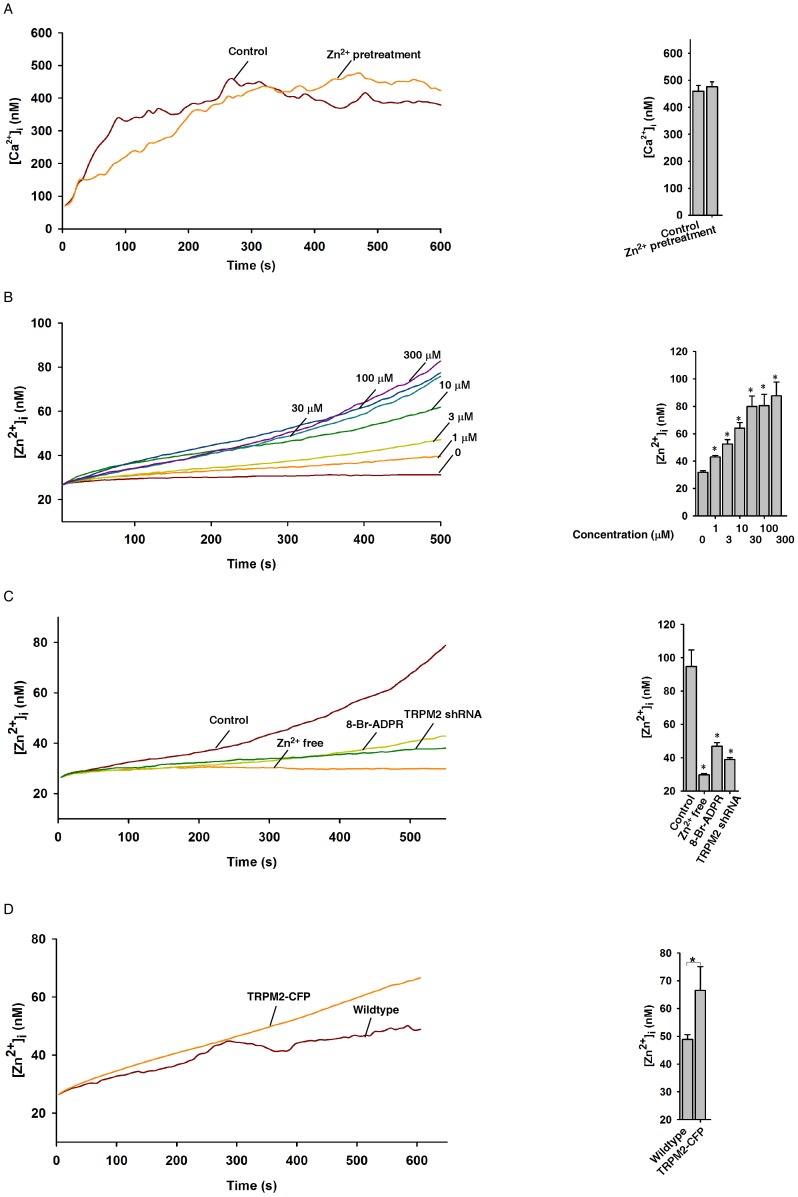
Characterization of the NPE-ADPR induced Zn^2+^ influx. (**A**) Pretreatment of Jurkat cells with Zn^2+^ did not affect the ability of the photolyzed NPE-ADPR (30 µM) to induce Ca^2+^ influx in Fluo-4 loaded human Jurkat cells. (**B**) Uncaging of intracellular NPE-ADPR induced intracellular Zn^2+^ increases in a concentration dependent manner in FluoZin-3 loaded human Jurkat cells. The * symbols indicate the results of *t* Test analysis, *p*<0.05, compared with cells loaded with buffer only. (**C**) Pretreatment of FluoZin-3 loaded Jurkat cells with 8-Br-ADPR (100 µM), or knockdown of TRPM2, or removal of extracellular Zn^2+^ markedly inhibited uncaged NPE-ADPR (100 µM)-induced Zn^2+^ influx in FluoZin-3 loaded human Jurkat cells. The * symbols indicate the results of *t* Test analysis, *p*<0.05, compared with cells without pretreatment. (**D**) Uncaging of NPE-ADPR induced Zn^2+^ influx in FluoZin-3 loaded HEK293 cells that transiently express TRPM2-CFP. Data quantifications of [Zn^2+^]_i_ peak induced by drug treatment in (B), (C), and (D) were expressed as mean ± S.E., n = 30–40 cells. In (A), (B), (C), and (D), after Fluo-4 or FluoZin-3 loaded cells were incubated with NPE-ADPR, extracellular NPE-ADPR was then removed before UV photolysis to start Ca^2+^ or Zn^2+^ measurement.

It has been previously shown that extracellular Zn^2+^ can inhibit ADPR-induced cation entry via TRPM2 [41]. However, we found that high concentrations of Zn^2+^ were toxic to both Jurkat and HEK 293 cells (data not shown). We therefore examined the effects of non-toxic concentrations of extracellular Zn^2+^ on TRPM2 in Jurkat cells. Surprisingly, extracellular Zn^2+^ at low concentrations had little effect on the ability of photolyzed NPE-ADPR to induce intracellular Ca^2+^ increases in Jurkat cells (Figures 5A).

We then examined whether photolyzed NPE-ADPR can directly induce Zn^2+^ influx via TRPM2 in Jurkat cells. The fluorescent intensity of FluoZin-3 loaded cells was used to indicate intracellular Zn^2+^ concentration. Interestingly, photolyzed NPE-ADPR induced intracellular Zn^2+^ increases in a dose dependent manner (Figure 5B), which was abolished by removal of extracellular Zn^2+^, pretreatment with 8-Br-ADPR, or knockdown of TRPM2 (Figure 5C). Not surprising, NPE-ADPR did not evoke any Zn^2+^ changes in Jurkat cells without UV uncaging, and UV illumination in the absence of NPE-ADPR also failed to induce Zn^2+^ (Figure S7). Consistently, uncaging of NPE-ADPR induced Zn^2+^ entry only in HEK 293 cells that overexpress TRPM2 but not in wildtype cells (Figure 5D). In summary, our data supported that uncaging of NPE-ADPR induces Zn^2+^ entry via TRPM2 as well.

### The Effects Of Temperature And Ph On Npe-Adpr Induced Cation Entry

**Figure 6:**
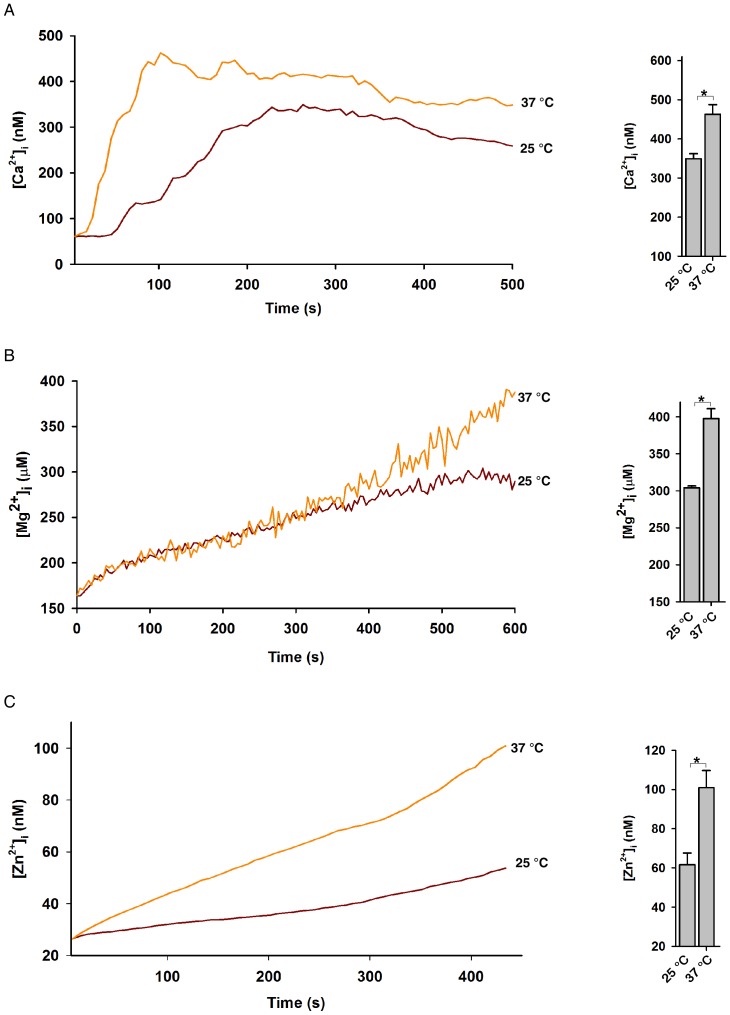
The effect of temperature on cation entry in Jurkat cells after uncaging of NPE-ADPR. (**A**), (**B**), **and** (**C**) The photolyzed NPE-ADPR (30 µM)-induced increases of intracellular Ca^2+^ (A), Mg^2+^ (B), and Zn^2+^ (C) were enhanced in high temperature in human Jurkat cells in regular HBSS. In (A), (B), and (C), after dye loaded cells were incubated with NPE-ADPR, extracellular NPE-ADPR was then removed before UV photolysis to start measurement. Data quantifications of peak induced by drug treatment in (A), (B), and (C) were expressed as mean ± S.E., n = 30–40 cells. The * symbols indicate the results of *t* Test analysis, *p*<0.05.

**Figure 7:**
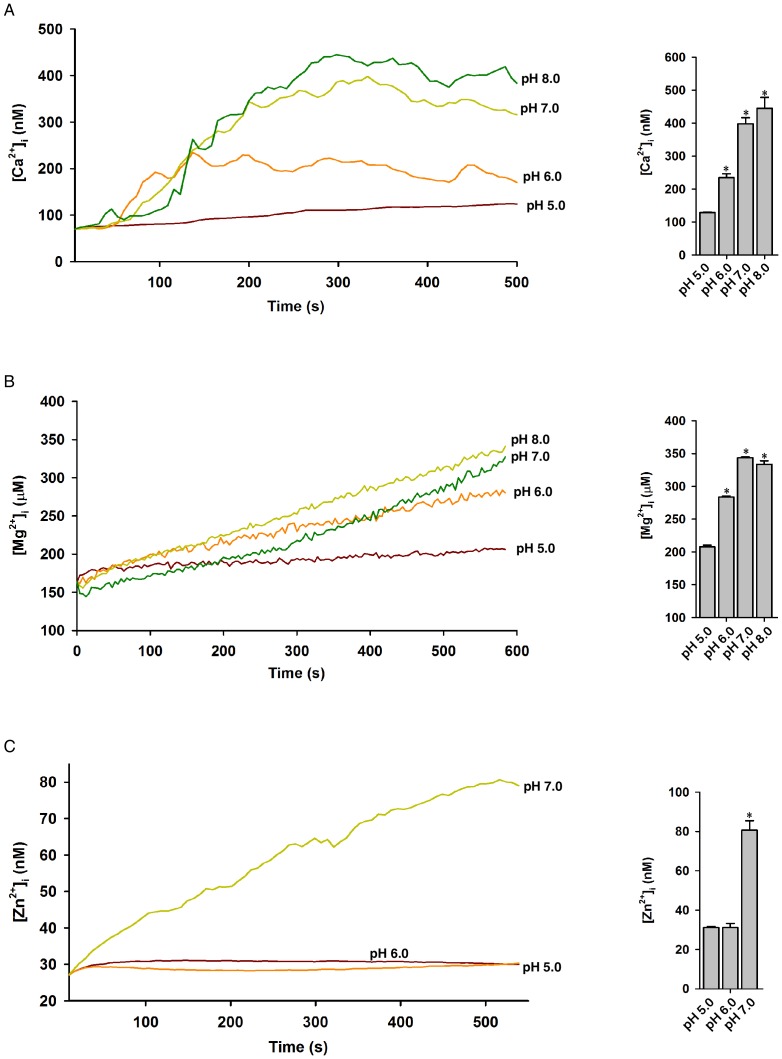
The effect of pH on cation entry in Jurkat cells after uncaging of NPE-ADPR. (**A**), (**B**), **and** (**C**) The photolyzed NPE-ADPR (30 µM)-induced increases of intracellular Ca^2+^ (A), Mg^2+^ (B), and Zn^2+^ (C) were inhibited by acidic pH in human Jurkat cells in regular HBSS. In (A), (B), and (C), after dye loaded cells were incubated with NPE-ADPR, extracellular NPE-ADPR was then removed before UV photolysis to start measurement. Data quantifications of peak induced by drug treatment in (A), (B), and (C) were expressed as mean ± S.E., n = 30–40 cells. The * symbols indicate the results of *t* Test analysis, *p*<0.05, compared with cells incubated in pH 5.0.

It has been previously reported that TRPM2 gating is modulated by pH and temperature [8],[31]–[35]. Indeed, we found that the abilities of photolyzed NPE-ADPR to induce the increases of intracellular Ca^2+^ (Figure 6A), Mg^2+^ (Figure 6B), or Zn^2+^ (Figure 6C) were much higher at 37°C than those at 25°C. Likewise, acidic pH (<7) markedly inhibited the photolyzed NPE-APDR from inducing the increases of intracellular Ca^2+^ (Figure 7A), Mg^2+^ (Figure 7B), and Zn^2+^ (Figure 7C) as compared to neutral (∼7) or alkaline (>8) pH in Jurkat cells. It is noteworthy that the photolyzed NPE-ADPR induced Ca^2+^ increase was higher in alkaline pH compared to that in neutral pH, which is most likely due to the fact that alkaline pH additionally inhibits SERCA to induce an intracellular Ca^2+^ rise [42]. Nevertheless, these data clearly indicated that temperature and pH modulate the gating of TRPM2 by ADPR.

### The Effects Of Extracellular Cations On Npe-Adpr Induced Cation Entry

**Figure 8:**
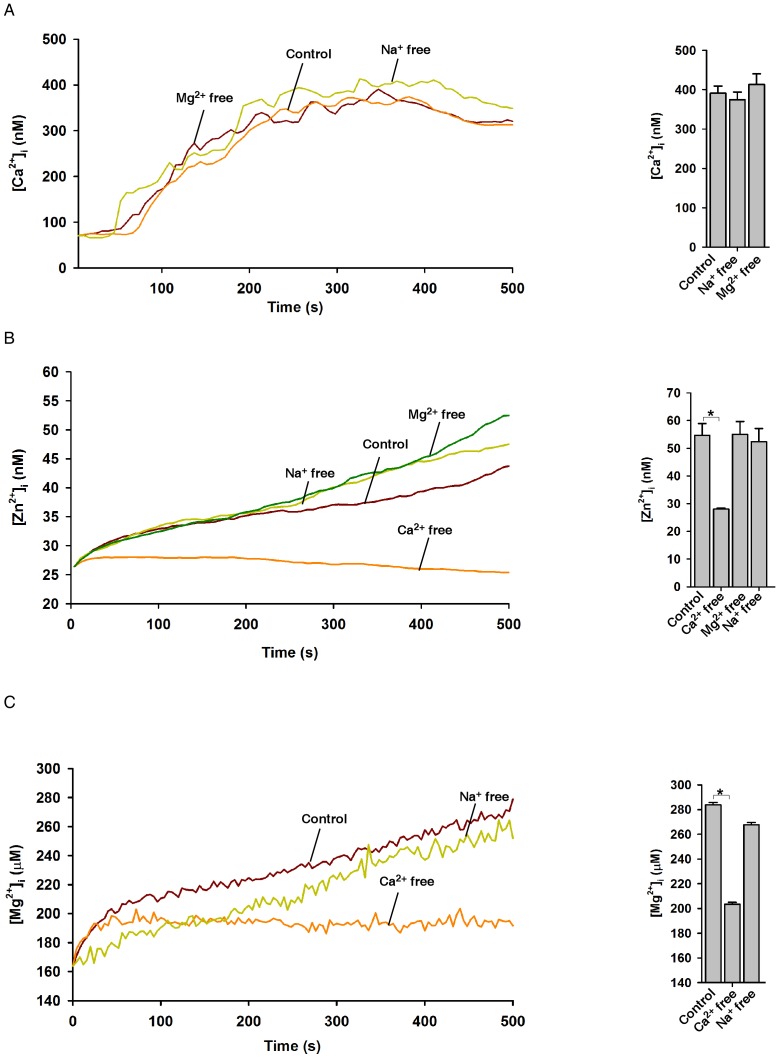
The effects of extracellular cations on NPE-ADPR induced cation entry in Jurkat cells. (**A**) The effects of varied cation compositions on uncaged NPE-ADPR induced Ca^2+^ influx in Fluo-4 loaded human Jurkat cells. (**B**) The effects of varied cation compositions on uncaged NPE-ADPR induced Zn^2+^ influx in FluoZin-3 loaded human Jurkat cells. (**C**) The effects of varied cation compositions on uncaged NPE-ADPR induced Mg^2+^ influx in Mag-Fura-2 loaded human Jurkat cells. In (A), (B), and (C), extracellular NPE-ADPR was removed before UV photolysis to start Ca^2+^, Zn^2+^, or Mg^2+^ measurement. Data quantifications of peak induced by drug treatment in (A), (B), and (C) were expressed as mean ± S.E., n = 30–40 cells. The * symbols indicate the results of *t* Test analysis, *p*<0.05.

Although ADPR is the primary activator for TRPM2 gating, intracellular Ca^2+^ was also implicated as an important modulator for TRPM2. We, therefore, examined the effects of varied extracellular cation compositions on photolyzed NPE-ADPR induced cation entry (Table 1). As shown in Figure 8A, the absence of extracellular Na^+^, Mg^2+^, or Zn^2+^ had no effect on photolyzed NPE-ADPR induced Ca^2+^ entry. On the other hand, the absence of extracellular Ca^2+^ not only abolished the induced Ca^2+^ influx (Figure 3A), but also markedly inhibited the induced Zn^2+^ (Figure 8B) or Mg^2+^ (Figure 8C) influx. The absence of extracellular Na^+^ or Mg^2+^ had no effect on the induced Zn^2+^ influx (Figure 8B), and the absence of extracellular Na^+^ also had no effects on induced Mg^2+^ influx (Figure 8C). In summary, these data indicated that extracellular Ca^2+^ is important for ADPR to activate TRPM2 for cation entry, possible by changing intracellular Ca^2+^ concentration via influx.

**Table 1:** Composition of different test solutions (in mM).

Solutions (A–L)	CaCl_2_	MgCl_2_	MgSO_4_	KCl	KH_2_PO_4_	NaHCO_3_	NaCl	Na_2_HPO_4_	ZnSO_4_	NMDG-Cl^1^	D-Glucose
A.Standard HBSS^2^	1.26	0.493	0.407	5.33	0.441	4.17	137.93	0.338	0	0	5.56
B. Ca^2+^ free HBSS^3^	0	0	0	5.33	0.441	4.17	137.93	0.338	0	0	5.56
C. Mg^2+^ buffer^4^	1.26	0.493	0.407	5.33	0.441	4.17	137.93	0.338	0	0	5.56
D. Mg^2+^ free buffer^5^	2.16	0	0	5.33	0.441	4.17	137.93	0.338	0	0	5.56
E. Zn^2+^ buffer^6^	1.26	0.493	0.407	5.33	0.441	4.17	137.93	0.338	0.03	0	5.56
F. Zn^2+^ free buffer^7^	1.26	0.493	0.407	5.33	0.441	4.17	137.93	0.338	0	0	5.56
G. Ca^2+^ free buffer^8^	0	0.493	0.407	5.33	0.441	4.17	137.93	0.338	0	0	5.56
H. Ca^2+^ free buffer^9^	0	0	0	5.33	0.441	4.17	137.93	0.338	0.03	0	5.56
I. Mg^2+^ free buffer^10^	2.16	0	0	5.33	0.441	4.17	137.93	0.338	0	0	5.56
J. Mg^2+^ free buffer^11^	2.16	0	0	5.33	0.441	4.17	137.93	0.338	0.03	0	5.56
K. Na^+^ free buffer^12^	1.26	0.493	0.407	5.33	0.441	0	0	0	0	142.7	5.56
L. Na^+^ free buffer^13^	1.26	0.493	0.407	5.33	0.441	0	0	0	0.03	142.7	5.56

1 : N-methyl-D-glucamine chloride;

2 : Invitrogen, 14025;

3 : Invitrogen, 14175;

4,5 : Mg^2+^ (+/−) buffer for magnesium measurement;

6,7 : Zn^2+^ (+/−) buffer for zinc measurement;

8 : Ca^2+^ (−) buffer for magnesium measurement;

9 : Ca^2+^ (−) buffer for zinc measurement;

10 : Mg^2+^ (−) buffer for calcium measurement;

11 : Mg^2+^ (−) buffer for zinc measurement;

12 : Na^+^ (−) buffer for calcium and magnesium measurement;

13 : Na^+^ (−) buffer for zinc measurement.

doi:10.1371/journal.pone.0051028.t001

## Discussion

Here we reported the synthesis and characterization of a NPE-caged ADPR. We found that the compound is permeable to Jurkat cells and HEK293 cells. Uncaging of intracellular NPE-ADPR induced the entry of multiple cations, including Ca^2+^, Mg^2+^, and Zn^2+^, via TRPM2. Thus NPE-ADPR is a useful cell permeant ADPR analogue and can be used to study the mechanisms of TRPM2-mediated cation entry.

NPE-ADPR is biologically inert before photolysis, suggesting that the phosphate groups are important for TRPM2 gating. Interestingly, the ester linkage between NPE and the phosphate is prone to UV photolysis, whereas the ether linkage between NPE and the hydroxyl group of ribose is relatively stable and resistant to UV photolysis (Figures 2A and 2B). These data indicated that ester bond has higher hydrolytic ability than ether bond under physiological condition. We speculate that the NPE group nearing the acidic phosphate could be easily protonated, thereby tending to be photolyzed more efficiently [43].

We showed in this study that attaching a cage group to the phosphate of ADPR can greatly increase its cell permeability (Figure S8), presumably because of the reduction of the charge of the phosphate as well as the increased lipophilicity contributed by NPE group. To minimize the leakage of the loaded NP-ADPR after washing, the photolysis was performed promptly after loading. Nevertheless, we observed a small Ca^2+^ increases in wildtype HEK293 cells lacking TRPM2 after photolysis (Figure 3E), which might be due to leakage of the probe and its activation of the P2Y1 receptor after photolysis. Indeed, treating cells with a P2Y1 inhibitor, suramin, can almost completely block the effects of extracellular uncaged NPE-ADPR on P2Y1 (Figures 3A and S3).

Interestingly, except Ca^2+^, neither Mg^2+^ nor Na^+^ had no significant effects on TRPM2 gating by ADPR. It has been shown previously that endogenous Ca^2+^ activates TRPM2 via its N-terminal calmodulin binding IQ-like motif possibly through calmodulin interaction [44],[45]. Thus, the effect of absence of extracellular Ca^2+^ on TRPM2 gating is likely due to the decrease intracellular Ca^2+^ concentration. Yet, it remains to be determined whether extracellular Ca^2+^ regulates some residues in the outer pore of TRPM2 for its activation.

Our results, in agreement with a patch clamp study [40], clearly demonstrated that ADPR can activate TRPM2 for Mg^2+^ influx. Mg^2+^ is one of the most abundant intracellular divalent cations and has been proposed to be able to serve as an intracellular second messenger, in addition to its well-known role of being a cofactor to ATP and a variety of enzymes [46]. Since a variety of stimuli can induce the generation of endogenous ADPR to incite intracellular Ca^2+^ increases via TRPM2 [11],[22]–[24],[47], these stimuli may well activate TRPM2 to cause not only Ca^2+^ influx but Mg^2+^ influx as well. In addition, TRPM2 mutants have been associated with several neurological diseases. These mutants led to decreases in Ca^2+^ influx [17]–[20]. In this regard, the role of Mg^2+^ in the neurological diseases associated with mutations in TRPM2 may well be worth re-examining [17]–[20]. The ease to monitor Mg^2+^ influx using NPE-ADPR as described in this study should facilitate this kind of investigations.

Likewise, the tool developed in this study should benefit investigations of the role of Zn^2+^ as an important regulator implicated in diverse cellular processes. Indeed, we show that uncaged NPE-ADPR can induce Zn^2+^ influx via TRPM2 in the presence of low concentration of extracellular Zn^2+^. Intracellularly, Zn^2+^ not only serves as an allosteric ion for transcription factors and metabolic enzymes, but also can modulate a variety of ion channels in a concentration dependent manner [48]–[50]. For example, TRPM3 and TRPM7 are both Zn^2+^ permeable [51],[52], while Zn^2+^ reversibly inhibits TRPM1 [53]. Similarly, high concentration of extracellular Zn^2+^ inhibited the ability of ADPR to activate TPRM2 for cation entry [41]. Here we showed that the absence or a low concentration of extracellular Zn^2+^ had no inhibitory or enhancive effects on TPRM2 gating. Future work is required to assess whether extracellular stimuli could change intracellular Zn^2+^ concentration via ADPR/TRPM2, and to determine the residues responsible for Zn^2+^ passage in TRPM2.

## Materials And Methods

### Chemistry

All of the chemical reagents were purchased from Sigma. The caged group 1-(2-nitrophenyl) diazoethane (compound **3**) was first synthesized as described previously [54]. Next ADPR (compound **4**, 50 mg, 0.089 mmol) dissolved in 3 mL ice-cold water was mixed with 3 mL 1-(2-nitrophenyl) diazoethane (compound **3**) dissolved in diethyl ether. The resulting biphasic mixture was vigorously stirred at 4°C in darkness for 3 h, and subsequently the ether layer was drawn off. 3 mL diazoethane reagent treatment procedure was then repeated three more times. Finally, purification of the water layer was performed by HPLC on a C18 reversed phase column, eluting with a linear gradient of 0–30% CH_3_CN in water within 30 min. Four peaks were collected, and peak A gave rise to the light yellow solid compound **5** (15.2 mg, 24%). According to the ^1^H NMR spectrum, this caged structure represented a mixture of more than one mono-caged isomers (Figure 1A). They were all efficiently photolyzed into ADPR under UV flash as detected by HPLC analysis (Figure 2A). ^1^ H NMR (400 MHz, DMSO-*_d6_*) δ8.42, 8.17 (s, each 1 H), 8.0-7.0 (m, 4 H), 5.92 (d, 1 H, *J* = 4 Hz), 4.93 (m, 1 H), 4.56 (m, 1 H), 4.23-4.18 (m, 2 H), 4.07-3.97 (m, 4 H), 1.24 (d, 3 H, *J* = 8 Hz). ^31^P NMR (100 MHz, DMSO-*_d6_*) δ-1.22, -2.04 ppm (Figures S1A and 1B). High resolution mass spectrometry (electrospray ionization, negative) for C_23_H_30_N_6_O_16_P_2_, calculated 707.1194 [M-1]^−^, found 707.1120.

In addition, peak D was collected and characterized as a caged structure containing five NPE groups (Figure 1A). ^1^ H NMR (400 MHz, DMSO-*d_6_*) δ8.40, 8.16 (s, each 1 H), 7.88-7.48 (m, 20 H), 5.92 (d, 1 H, *J* = 4 Hz), 5.11 (q, 5 H, *J* = 8 Hz), 4.93 (m, 1 H), 4.56 (m, 1 H), 4.27-4.23 (m, 2 H), 4.06-3.98 (m, 4 H), 1.37 (d, 15 H, *J* = 8 Hz). ^31^P NMR (100 MHz, DMSO-*d_6_*) δ-11.03, -11.25 ppm.

8-Br-ADPR was synthesized and purified as described previously [38].

### Cell Culture

The human Jurkat T-lymphocytes and human embryonic kidney (HEK) 293 cells were both obtained from *ATCC* (Manassas, VA). Jurkat cells were normally cultured in RPMI medium 1640 (Invitrogen) supplemented with 10% fetal bovine serum (FBS), 100 units/ml penicillin/streptomycin, and 2 mM Hepes buffer (pH 7.4) at 5% CO_2_ and 37°C. HEK293 cells were cultured in DMEM medium (Invitrogen) supplemented with 10% FBS and 100 units/ml penicillin/streptomycin at 5% CO_2_ and 37°C.

### Transient Transfection

HEK293 cells were plated at a density of 3×10^5^ cells/well in 6-well plates. On the next day, 2 hours before transfection, the medium was changed to an antibiotic-free medium. The pCI-CFP-hTRPM2 or empty vector pCI-CFP was then transfected into cells by Lipofectamine™ 2000 (Invitrogen). 24 hours after transfection, the medium was changed to regular medium, and TRPM2-CFP or CFP positive cells were finally used for Ca^2+^, Zn^2+^, or Mg^2+^ measurement after another 24 hours.

### Imaging Measurements

Imaging measurements were performed as described previously [42],[55],[56]. Jurkat cells (2×10^5^ cells/well) or HEK293 cells (6×10^4^ cells/well) were plated in 24-well plates coated with 100 or 10 µg/ml poly-L-lysine (Sigma, P6282) respectively and both were incubated in serum free medium at 37°C overnight for adherence. The adherent cells were incubated with 2 µM Fluo-4 AM (Invitrogen, F14201), or FluoZin-3 AM (Invitrogen, F24195), or Mag-Fura-2 (Invitrogen, M1292) in Hanks' balanced salt solution (HBSS) in darkness at 37°C. The cells were then washed with HBSS twice and incubated in 200 µL of different test solutions (A–L, Table 1). Thereafter, the cells were put on the stage of an Olympus inverted epifluorescence microscope and incubated with or without caged ADPR in the presence or absence suramin (# 574625, CalBiochem) for 60 min followed by UV (370 nm) flash for 1 s to photolyze the caged structure, which was repeated every 7 s during the measurement of fluorescence intensity at 480 nm for Fluo-4 and FluoZin-3 using a 20× objective. Images were collected by a CCD camera every 7 s and analyzed by the cell R imaging software. For Mag-Fura-2 measurements, fluorescence was measured using the same imaging system, operating in ratio mode with emission set at 510 nm and alternating excitation at 340 and 380 nm every 4 s. For the measurements under different temperatures, an incubation system (Olympus, MIU-IBC) was applied.

### Data Analysis

In each measurement, intracellular concentration of calcium, zinc, or magnesium was calculated using the general formula, [Ca^2+^]*_i_* = *K_d_*(*F*-*F*_min_)/(*F*_max_-*F*) (*K_d_* = 345 nM), [Zn^2+^]*_i_* = *K_d_*(*F*-*F*_min_)/(*F*_max_-*F*) (*K_d_* = 15 nM), or [Mg^2+^]*_i_* = *K_d_*(*R*-*R*_min_)/(*R*_max_-*R*) (*K_d_* = 1.45 mM), respectively. *K*_d_ is the dissociation constant for Ca^2+^ or Mg^2+^ or Zn^2+^ binding to the indicator, F is the fluorescence intensity with Fluo-4 or FluoZin-3, and R is the ratio between emission at 340 and 380 nm with Fura-2. For Fluo-4, *F*_max_ was determined by exposing cells to 10 mM Ca^2+^ and 5 µM ionomycin, and *F*_min_ was determined by the addition of 4 mM EGTA and 5 µM ionomycin to cells. For FluoZin-3, *F*_max_ was determined by exposing cells to 1 mM Zn^2+^ and 20 µM pyrithion, and *F*_min_ was determined by the addition of 50 µM TPEN (N, N, N′, N′-tetra- (2-picolyl) ethylenediamine) and 20 µM pyrithion to cells. For Mag-Fura-2, *R*_max_ was determined by exposing cells to 30 mM Mg^2+^ and 5 µM ionomycin, and *R*_min_ was determined by the addition of 10 mM EGTA and 5 µM ionomycin to cells. Significant differences of peak ion level between groups were determined by the Student's *t* test, in which * p<0.05 was validated to be significant.

## Supporting Information

Figure S1 NPE-ADPR (30 µM) did not evoke any Ca2+ changes in Jurkat cells without UV uncaging, and UV illumination in the absence of NPE-ADPR also failed to induce Ca^2+^. (PDF)

Figure S2 Direct application of ADPR to the medium induced cytosolic Ca^2+^ increase in Fluo-4 loaded human Jurkat cells incubated in the regular HBSS (purple line), and uncaging of NPE-ADPR induced cytosolic Ca^2+^ increase in Fluo-4 loaded Jurkat cells in the absence of external Ca^2+^ (orange line). (PDF)

Figure S3 Combination of suramin with 8-Br-ADPR or TRPM2 knockdown completely blocked the photolyzed NPE-ADPR (30 µM) induced [Ca2+]_i_ increases in Jurkat cells. The Fluo-4 loaded Jurkat cells in the regular HBSS were continuously incubated with NPE-ADPR throughout the experiments. (PDF)

Figure S4 The DIC and fluorescence images of HEK293 cells that transiently express TRPM2-CFP. (PDF)

Figure S5 NPE-ADPR (300 µM) did not evoke any Mg^2+^ changes in Jurkat cells without UV uncaging, and UV illumination in the absence of NPE-ADPR also failed to induce Mg^2+^. (PDF)

Figure S6 The anti-CD3 antibody, OKT3 (2 µg/ml), markedly induced Ca^2+^ increases in Fura-2 loaded Jurkat cells, whereas it failed to induce any fluorescence changes on Maga-Fura-2 loaded cells. (PDF)

Figure S7 NPE-ADPR (100 µM) did not evoke any Zn^2+^ changes in Jurkat cells without UV uncaging, and UV illumination in the absence of NPE-ADPR also failed to induce Zn^2+^. (PDF)

Figure S8 The Jurkat cells were incubated with NPE-ADPR or ADPR in regular HBSS for 5 min. The concentrations of NEP-ADPR or ADPR in HBSS before and after incubation were measured by UV absorbance (265 nM) and subsequently calibrated against respective standard concentration curves. Data were expressed as mean ± S.D. from three independent experiments. The * symbols indicate the results of *t* Test analysis, *p*<0.05. (PDF)
