# Supplementary figures and images for: A Cell Permeable NPE Caged ADP-Ribose for Studying TRPM2
Source: PLoS One. 2012 Dec 7;7(12):e51028. doi: 10.1371/journal.pone.0051028 (PMC3517590; doi:10.1371/journal.pone.0051028)

Figure S1

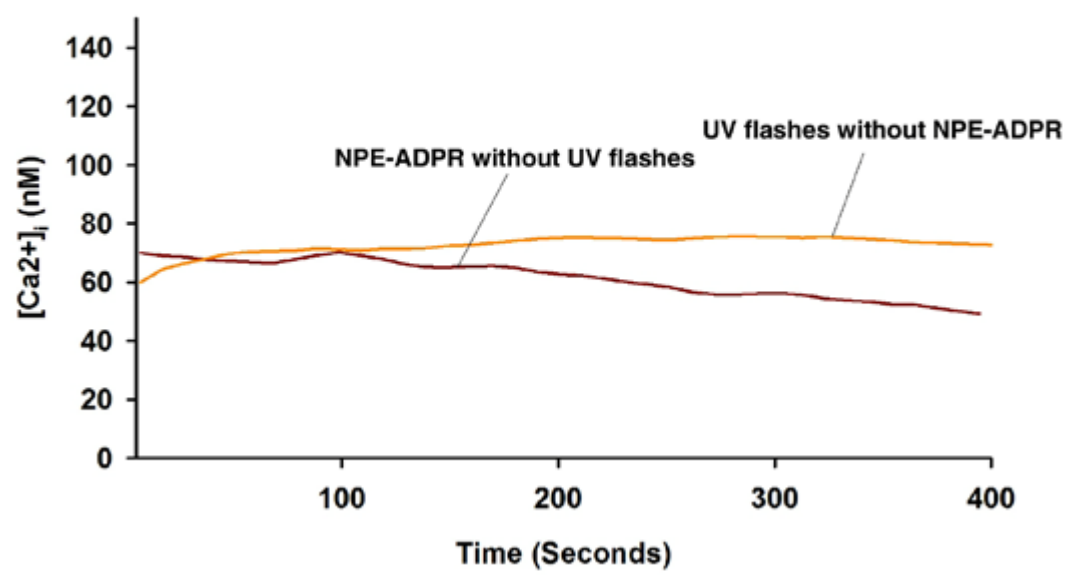

Supplement: Figure S1 — (PDF) [file pone.0051028.s001.pdf]

Figure S2

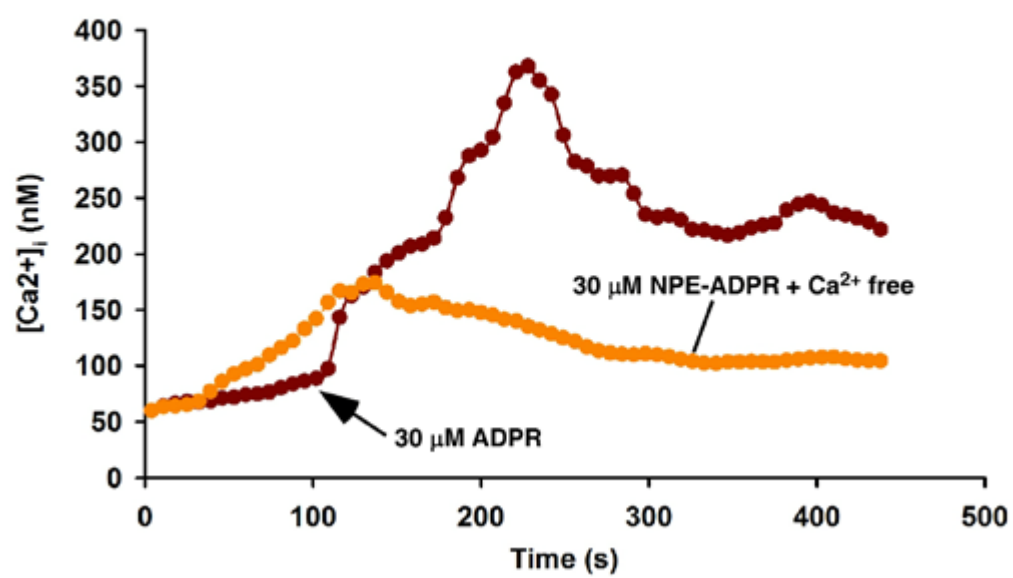

Supplement: Figure S2 — (PDF) [file pone.0051028.s002.pdf]

Figure S3

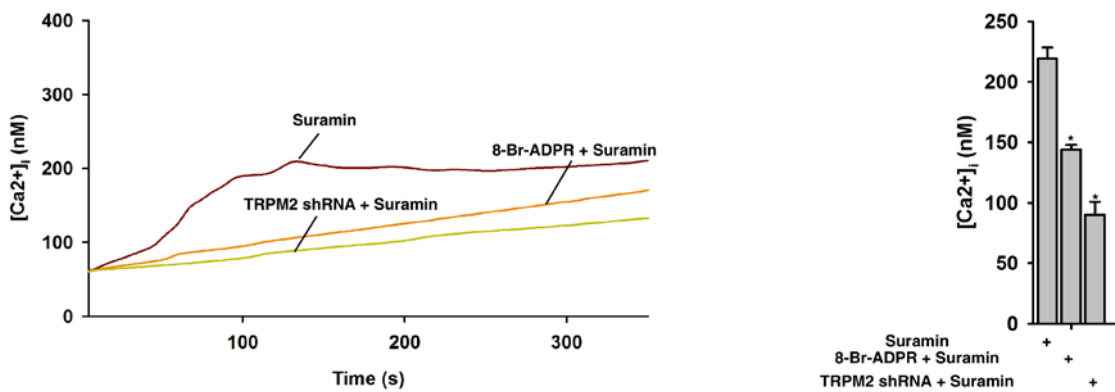

Supplement: Figure S3 — (PDF) [file pone.0051028.s003.pdf]

Figure S4

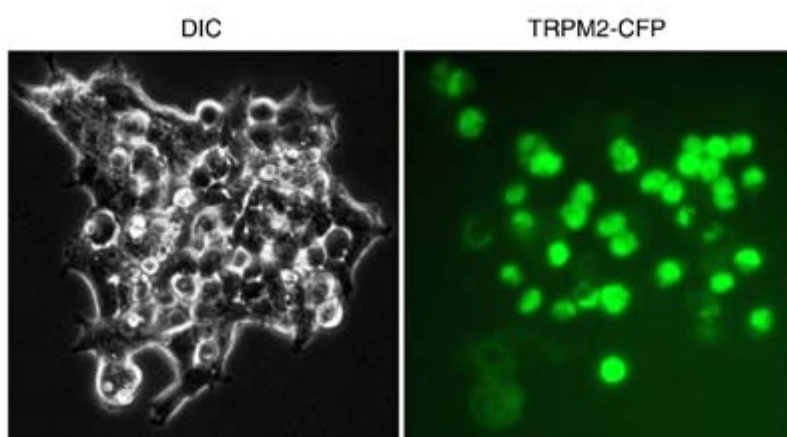

Supplement: Figure S4 — (PDF) [file pone.0051028.s004.pdf]

Figure S5

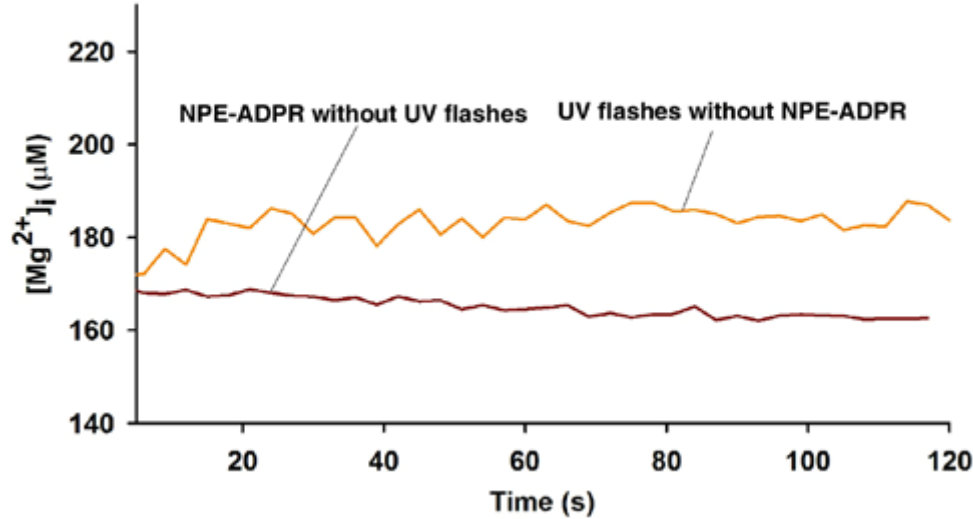

Supplement: Figure S5 — (PDF) [file pone.0051028.s005.pdf]

Figure S6

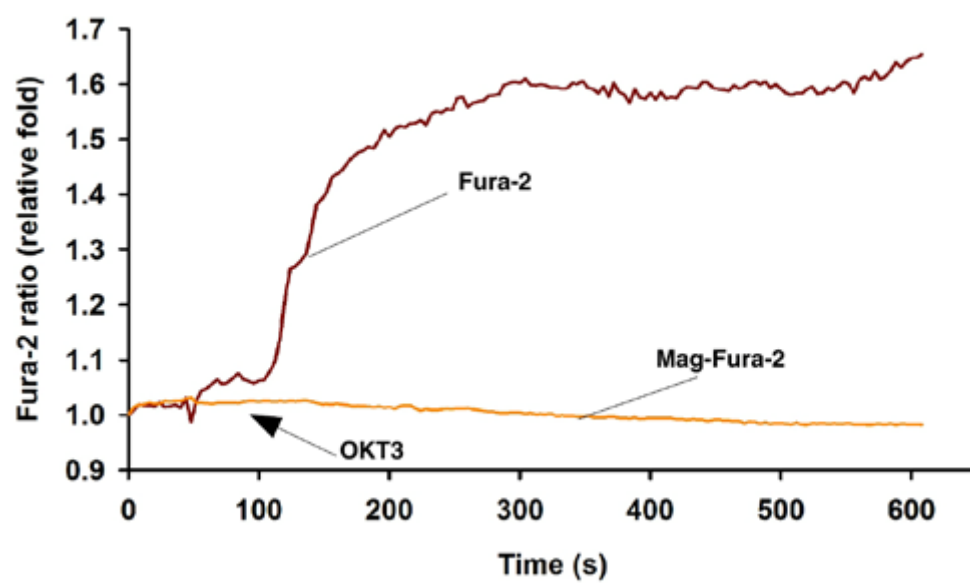

Supplement: Figure S6 — (PDF) [file pone.0051028.s006.pdf]

Figure S7

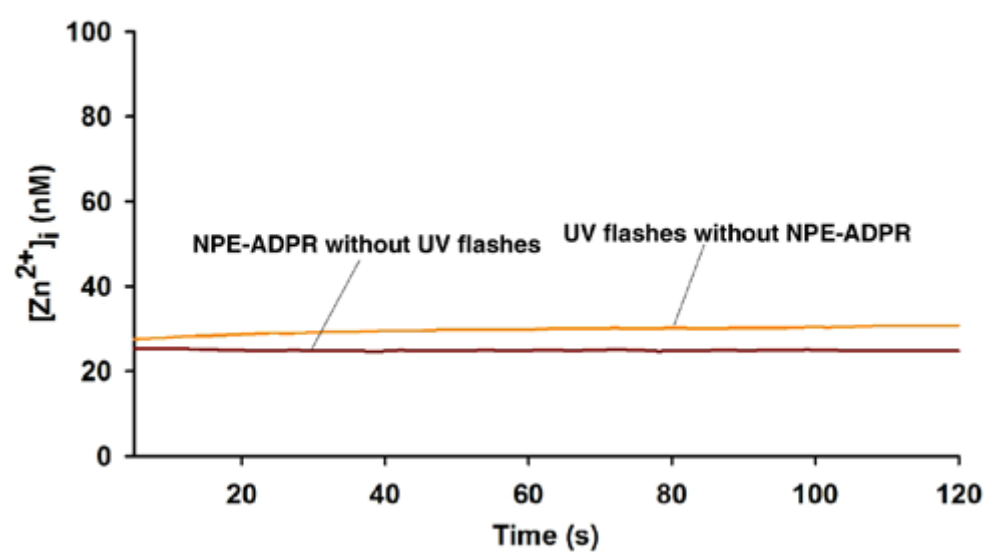

Supplement: Figure S7 — (PDF) [file pone.0051028.s007.pdf]

Figure S8

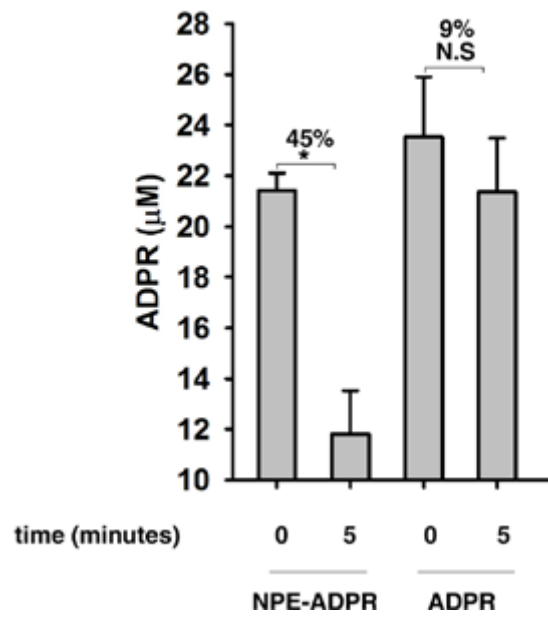

Supplement: Figure S8 — (PDF) [file pone.0051028.s008.pdf]
